# Specific and Evolving Resting-State Network Alterations in Post-Concussion Syndrome Following Mild Traumatic Brain Injury

**DOI:** 10.1371/journal.pone.0065470

**Published:** 2013-06-06

**Authors:** Arnaud Messé, Sophie Caplain, Mélanie Pélégrini-Issac, Sophie Blancho, Richard Lévy, Nozar Aghakhani, Michèle Montreuil, Habib Benali, Stéphane Lehéricy

**Affiliations:** 1 Inserm, UPMC Univ Paris 06, UMRS 678, Laboratoire d’Imagerie Fonctionnelle, Paris, France; 2 IFR49, Univ Paris 11, DSV/I2BM/NeuroSpin, Bât 145, Gif-sur-Yvette, France; 3 Vincennes-Saint-Denis Univ Paris 08, EA 2027, Psychopathologie et Neuropsychologie, Saint-Denis, France; 4 Institut pour la Recherche sur la Moelle Epinière et l’Encéphale, Paris, France; 5 Inserm, UPMC Univ Paris 06, UMRS 975, CNRS UMR 7225, CRICM, Groupe Hospitalier Pitié-Salpêtrière, Paris, France; 6 Assistance publique - Hôpitaux de Paris, Hôpital Saint-Antoine, Paris, France; 7 Assistance publique - Hôpitaux de Paris, Hôpital Universitaire Bicêtre, Neurosurgery department, Kremlin-Bicêtre, France; 8 Université de Montréal, MIC/UNF, Montréal, Canada; 9 UPMC Univ Paris 06, CHU Pitié-Salpêtrière, CENIR, Center for Neuroimaging Research, Paris, France; Cuban Neuroscience Center, Cuba

## Abstract

Post-concussion syndrome has been related to axonal damage in patients with mild traumatic brain injury, but little is known about the consequences of injury on brain networks. In the present study, our aim was to characterize changes in functional brain networks following mild traumatic brain injury in patients with post-concussion syndrome using resting-state functional magnetic resonance imaging data. We investigated 17 injured patients with persistent post-concussion syndrome (under the DSM-IV criteria) at 6 months post-injury compared with 38 mild traumatic brain injury patients with no post-concussion syndrome and 34 healthy controls. All patients underwent magnetic resonance imaging examinations at the subacute (1–3 weeks) and late (6 months) phases after injury. Group-wise differences in functional brain networks were analyzed using graph theory measures. Patterns of long-range functional networks alterations were found in all mild traumatic brain injury patients. Mild traumatic brain injury patients with post-concussion syndrome had greater alterations than patients without post-concussion syndrome. In patients with post-concussion syndrome, changes specifically affected temporal and thalamic regions predominantly at the subacute stage and frontal regions at the late phase. Our results suggest that the post-concussion syndrome is associated with specific abnormalities in functional brain network that may contribute to explain deficits typically observed in PCS patients.

## Introduction

Post-concussion syndrome (PCS) following mild traumatic brain injury (mTBI) remains one of the most elusive and challenging pathological conditions. PCS is characterized by the presence of subjective complaints such as fatigue, headaches, sleep disturbance or poor concentration [1]–[3], and induces substantial socio-professional troubles that may last from several months to years [4]–[6]. The degree to which PCS is associated with brain lesions is still debated. Several studies have suggested that cognitive and neurobehavioral disorders observed in patients with PCS may be associated with axonal injury [7]–[10]. Consequences of mTBI on large-scale brain networks and function in PCS patients remain to be investigated.

Large-scale neural networks are distributed local neural assemblies often linked by long-distance structural connections [11]. Brain networks are thought to form an essential substrate for the performance of most cognitive functions [12]. They can be explored using resting-state fMRI, i.e. in the absence of any explicit task. Resting-state fMRI enables an indirect access to intrinsic neuronal activity through its metabolic and hemodynamics consequences [13], [14], and has thus provided critical insights into brain function [15]–[17]. Resting-state fMRI networks can be characterized using graph theory, a mathematical framework allowing the exploration of the organizational topology of graphs [18]. Brain graphs are composed of sets of nodes, usually regions of variable size, linked by sets of edges, which can be either structural (white matter fiber tracts) or functional (dynamic synchronization). Graph theory studies have allowed neuroscientists to show that neural networks had efficient small-world properties [18]–[21] and rich-club organization [22]. Such architecture is suggested to satisfy the competitive demands of brain networks in local and global information processing [23], [24].

In patients, recent works have highlighted the consequences of mTBI on functional brain networks. Disrupted resting-state fMRI networks were evidenced at the acute to subacute phases of injury, specifically in the default mode network [25], [26]. Functional connectivity within the default mode network was correlated to cognitive impairment [27], [28], as well as the number of mTBI episodes [25] and the degree of consciousness in severe TBI patients [29]. Based on a graph theory approach, alterations of resting-state functional networks were observed in mild to severe TBI patients including an overall increase in connectivity strength and efficiency, with values returning to those observed in healthy adults over the course of recovery [30], whereas no change in functional connectivity was observed in mild TBI over a long-term recovery period [27], [31]. These data suggest that patterns of connectivity in functional networks are altered following mild TBI, with possible recovery within months. It remains unclear whether resting-state brain networks are differently affected in patients with PCS because the consequences of PCS on these networks have not yet been explored. As PCS may be associated with greater axonal injury [7]–[10], it was expected that PCS patients would present greater functional alterations in large-scale brain networks particularly in the default mode network.

The aim of the present study was to characterize changes in functional brain networks following mild traumatic brain injury in patients with PCS using resting-state fMRI data. We analyzed functional brain networks at the subacute and late stages after mTBI in patients developing persistent PCS compared with patients without PCS and healthy volunteers. Functional brain organizational topology was assessed using resting-state fMRI and graph theory. We hypothesized that patients with mTBI would present graph properties alterations, as already previously reported. We further hypothesized that patients with PCS would present specific and greater alterations in regions involved in cognitive functions or mood (characteristically altered in PCS) such as frontal regions. Such findings would argue in favor of the physiological origin of PCS–related chronic symptoms.

## Materials and Methods

### Ethics Statement

This study was approved by the local Ethics Committee of the Pitié-Salpêtrière Hospital (Paris, France). After a complete description of the study, written informed consent was obtained for all participants. No concern regarding the possibility of reduced capacity to consent on his/her own was voiced by the neuropsychologist (SC).

### Participants

Mild TBI was defined according to the Head Injury Interdisciplinary Special Interest Group of the American Congress of Rehabilitation Medicine [32]. Trauma-induced physiological disruption of brain function manifested by at least one of the following signs: loss of consciousness of less than 30 min, Glasgow Coma Scale (GCS) score between 13 and 15, post-traumatic amnesia of less than 24 hours, any alteration in mental state at the time of injury (confusion, disorientation…), transient focal neurological deficit. Non-inclusion criteria of mTBI included history of chronic alcohol or drug abuse, previous TBI, contraindications to MRI, intubation and/or presence of a skull fracture and administration of sedatives on arrival in the emergency department, spinal cord injury, neurological signs or multiple disabilities (including at least one life-threatening injury associated), head injury following autolysis, patients with psychiatric or psychological disabilities that may interfere with the evaluation, psychotropic medication at the time of TBI, pre-existing neurological condition, major depressive syndrome. Exclusion criteria were patients not fully participating in the procedure, MRI artifacts or poor image quality (see next section).

A total of 96 consecutive patients with mTBI were initially recruited from the Emergency Department of Bicêtre (Kremlin-Bicêtre, France) and Bichat Hospitals (Paris, France). Out of them, 27 were excluded as they did not fulfill the entire procedure and 14 because of poor image quality (excessive head motion in 6 patients and incomplete examination in 8 patients). A total of 55 mTBI patients were finally included in the study (18 female; mean age 34.9±11.5 years). Healthy volunteers with no history of neurological or psychiatric disease, no contraindications for MRI, and no mTBI inclusion criteria were also recruited from the local community. From the 40 healthy volunteers initially recruited (11 female; mean age 36.8±12.6 years), 6 were excluded because of poor MRI quality (excessive head motion in 2 subjects and incomplete examination in 4 subjects). Patient and control subjects were matched for age, gender, and socio-cultural level (SCL, function of the number of years of education, ranging from 1 (primary school or less) to 5 (university degree), through 3 (Bachelor) [33]) (Table 1).

**Table 1:** Clinical and overall graph characteristics of all participants.

Characteristics				Controls	mTBI patients			
					Subacute phase		Late phase	
					PCS–	PCS+	PCS–	PCS+
**Clinic**		**Number**		34	38	17	–	–
		**Age**		36.8 (12.6)	34.2 (12.4)	36.4 (9.5)	–	–
		**Gender (F/M)**		11/23	11/27	7/11	–	–
		**SCL**		3.6 (1.2)	3.6 (1.1)	3.0 (1.4)	–	–
		**GCS**		–	15.0 (0.2)	14.9 (0.2)	–	–
		**Symptoms severity**		2.5 (2.6)	**9.8 (9.7)**	**31.1 (14.6)**	**3.6 (5.0)**	**25.5 (11.8)**
**Origins of injury**		**MVA**	**Car**		2	2	–	–
			**Motorbike**		2	2	–	–
			**Bicycle**		1	0	–	–
			**Pedestrian**		1	0	–	–
		**Falls**		–	4	5	–	–
		**Aggressions**		–	12	4	–	–
		**Work**		–	5	1	–	–
		**Sports**		–	6	0	–	–
		**Others**		–	5	3	–	–
**Graph properties**	**Basic**	**Cost**		0.53 (0.08)	0.52 (0.08)	0.51 (0.07)	0.53 (0.05)	0.51 (0.07)
		**Strength**		14.4 (3.4)	14.3 (3.3)	14.0 (2.5)	14.2 (2.1)	13.9 (2.6)
		**Edge diversity (10^−3^)**		33.8 (6.5)	33.7 (4.9)	33.6 (4.4)	33.9 (4.1)	33.5 (5.1)
		**Nodal diversity**		22.9 (11.3)	25.4 (16.6)	23.6 (11.2)	21.6 (8.3)	23.2 (12.7)
	**Topologic**	**Smallworld**		2.73 (0.22)	2.67 (0.16)	2.70 (0.19)	2.72 (0.16)	2.66 (0.14)
		**Global efficiency (10^−2^)**		50.2 (2.80)	50.1 (2.8)	50.2 (2.3)	50.7 (1.9)	50.3 (2.9)
		**Local efficiency (10^−2^)**		66.1 (4.0)	64.8 (2.3)	65.1 (3.0)	65.6 (2.9)	64.9 (2.6)
		**Modularity**		0.33 (0.05)	0.31 (0.05)	0.32 (0.04)	0.33 (0.04)	**0.31 (0.04)**
		**Hierarchy**		0.26 (0.04)	0.26 (0.05)	0.26 (0.04)	0.26 (0.03)	0.27 (0.04)
		**Assortativity**		0.24 (0.08)	0.25 (0.08)	0.25 (0.06)	0.25 (0.06)	0.25 (0.07)
		**Robustness**		2800 (151)	2788 (164)	2793 (140)	2823 (120)	2810 (156)

Abbreviations: GCS, Glasgow coma scale; F, female; M, male; MVA, motor vehicle accident; PCS, post-concussion syndrome; SCL, socio-cultural level. Mean and standard deviation (SD) values are given, except for gender. Bold indicates significance at p<0.05.

### Procedure

All patients underwent MRI investigation and clinical tests at the subacute (8–21 days) and late (6 months) phase after injury. Volunteers had only one MRI investigation and clinical test session. PCS was established at the late phase based on the DSM-IV criteria [34]. Patients with at least three post-concussion symptoms (even rated as mild) as well as objective neuropsychological impairment were classified as having persistent PCS (PCS+, PCS– otherwise). PCS symptoms included headaches, sleep disturbance, fatigue, dizziness, irritability, apathy, anxiety, depression or mood lability, personality change. In addition, PCS+ patients must have experienced social problems as a result, and did not present any other disorder that may have better explained the symptoms [35]. The post-concussion symptoms were assessed by the Rivermead Post-concussion Symptoms Questionnaire (RPSQ) [36], composed of 16 symptoms that commonly occur after brain injury (Table S1). Subjects rated the severity of each symptom within the last 24 hours from 0 (no or no more symptoms than after the trauma) to 4 (severe symptoms). Symptom severity was defined as the sum of scores over the 16 symptoms. Control subjects were given a slightly altered version of the RPSQ in which subjects were asked to rate the current incidence/problem-status for each of the 16 RPSQ items, but with no reference to a previous head injury.

### Data Acquisition and Preprocessing

The MRI protocol consisted of structural and functional images acquired using a 3T Siemens Trio TIM system (CENIR, ICM, Paris, France), with body coil for excitation and 12-channel head coil for signal reception. Structural images were obtained using a sagittal magnetization-prepared rapid gradient echo 3D T1-weighted sequence (field-of-view (FOV) 256×256 mm^2^; 176 contiguous slices; time repetition (TR)/echo time (TE)/time inversion (TI) = 2,300/4.18/900 ms; flip angle = 9°; voxel size 1 mm^3^ isotropic). Resting-state fMRI interleaved series were recorded using an axial echo-planar imaging sequence sensitive to blood oxygen level-dependent (BOLD) contrast (FOV 200×200 mm^2^; 38 contiguous slices; TR/TE = 2,650/30 ms, PAT = 2; voxel size 1.5×1.5×2 mm^3^). One hundred and eighty fMRI volumes were acquired during 10 min. The subjects were instructed to stay awake, eyes closed and motionless and to reduce any mental effort.

MRI data were preprocessed using the statistical parametric mapping software SPM5 (www.fil.ion.ucl.ac.uk/spm/software/spm5/). For each subject, the first 4 fMRI volumes were discarded to allow for T1 equilibration, and the remaining 176 volumes were corrected for slice-timing and head motion. Significant higher average temporal derivative displacement values (in translation and rotation) were found in PCS– patients at the subacute phase compared with controls (p = 0.005, permutation test). These metrics were further considered in statistical analyses to counteract possible effect on resulting graph properties. The resulting data were then spatially smoothed using an isotropic Gaussian kernel (full-width-at-half-maximum: 6 mm). Spatial registration using a rigid transformation between the fMRI data and the anatomical volume was computed for each subject. Tissue segmentation and non-linear spatial normalization to the standard space of the Montreal Neurological Institute (MNI) were also computed from the T1-weighted anatomical volume of each subject using the unified procedure of SPM5. Finally, fMRI datasets were registered to the MNI standard space using the transformations previously calculated and resampled at 2 mm isotropic resolution. Resting-state fMRI data with misplaced FOV, excessive head motion (greater than 3 mm or 3° in any dimension), artifacts due to MR instability or any technical problems, were not considered in the subsequent analysis.

### Construction of the Functional Brain Network

Functional brain network was constructed from each subject by defining a set of regions of interest (ROIs, or nodes) connected by a set of links (or edges).

#### Nodes

Brains regions were defined using the Automated Anatomical Labeling (AAL) template [37]. Because of the limited number of slices in fMRI volumes and variable brain coverage across individuals, regions were omitted in further analysis if fMRI data were only available in less than 30% of their voxels for any subject. Thirty regions were therefore excluded, comprising bilateral cerebellar cortex and vermis, fusiform, inferior occipital and inferior temporal cortex, and middle temporal pole, and the cortex was finally parcellated into 82 regions. Then time series of all voxels within a given region were spatially averaged to form the representative intrinsic functional BOLD time course of that region. To remove spurious sources of variance, linear and quadratic drifts, motion parameters, averaged ventricular and white matter signals were regressed out. Furthermore, we performed global brain signal regression. Finally time series were low-pass filtered (<0.1 Hz) [38], [39].

#### Edges

Pairwise inter-regional correlations were estimated between the resting-state BOLD time series of all regions. The null hypothesis that a given correlation coefficient was zero was tested to remove non-significant edges and emphasize the network topology. To account for the numerous non-independent tests, the false discovery rate (FDR) approach was applied to correct for multiple comparisons at q <0.05 [40]. Then correlation coefficients with p values exceeding q level were set to zero, leading to individual undirected and weighted functional connectivity matrices ***W*** *= (w_ij_)*, where *w_ij_* represents the absolute correlation coefficient between region *i* and *j* within the network.

### Characteristics of Brain Network Organization Using Graph Theory

A functional brain network can be efficiently represented as a graph of *N* nodes connected by *M* edges, and investigated using graph theory [18]. In the present analysis, the AAL brain regions represented the 82 cortical and subcortical areas from which mean fMRI time series were sampled. The edges represented the correlations between these time series and were computed as described in the previous section. All graph properties were computed using MATLAB software (R2011a, The MathWorks Inc., Natick, MA) and the Brain Connectivity Toolbox (www.brain-connectivity-toolbox.net) [41], as well as in-house scripts.

#### Basic properties

Basic graph properties based on raw weighted functional matrices (***W***) included graph cost, nodal strength and diversities. The graph cost, *c*, corresponds to the graph's density, i.e. the proportion of actual connections regarding the total number of possible connections. The nodal strength, *s*, was computed as the sum of all connections for a given region. The edge diversity of a node, *d_e_*, was defined as the variance of the connection weight over connections, while the nodal diversity, *d_n_*, was defined as the variance of the nodal strength over nodes. Diversity measures have recently been applied to brain networks [42], [43]. They measure the homogeneity of the connectivity distribution from a given region or over regions: the higher the measures, the less homogeneous the connectivity. Globally, nodal strength and edge diversity were defined as the average over nodes of the regional measures.

#### Topological measures

Topological properties detected several aspects of brain networks such as smallworldness –*how specialized groups of nodes are interrelated*– [44], integration –*amount of information flow*– and segregation –*dense interconnected nodes with specialized task*– using efficiency [45], modularity [46], hierarchy [47], [42] and centrality (for edge or node) [48] measures, assortativity –*preferential associations of nodes with similar degree*– [46], and resilience to attacks –*how robust networks behave in response to either random or targeted lesions*– [49], [43]. The most simplistic measure, at the origin of most metrics, was the degree of a node, *k*, which was defined as the number of edges connecting the node within the graph. Since the majority of these metrics have been previously defined and widely used, definitions are given in supporting information File S1. These measures have been originally designed for unweighted graphs and were highly dependent on graph cost. Therefore, in order to analyze topological properties of brain functional networks, each correlation matrix were thresholded to create binary graphs described by their adjacency matrix *A*, where *a_ij_* = 1, if the absolute value of the correlation between nodes *i* and *j* (*w_ij_*) exceeds a given threshold value, 0 otherwise. To ensure that topological measures were mathematically comparable across subjects, a common network cost was required. The choice of a threshold clearly influenced the resulting network architecture; therefore functional connectivity matrices were thresholded successively over a range of network costs. Some topological measures such as smallworldness required that the mean degree of the graph was greater than *log(N)* [50], this defined the minimal possible cost value of 0.06 (with 82 regions), while the maximal available cost was given by the minimal cost of the raw functional connectivity matrices (*W*, here 0.31). Therefore, we defined the available cost range, 0.06–0.31, as cost values between the minimal and maximum cost available for all subjects under these conditions. Topological results reported in this study are thus averages of the various metrics estimated for each individual network over the available cost range. Such cost-integration approach has been found to be reliable for disentangling cost differences from topology differences [51], [52].

### Statistics and Visualization

Classical statistical inference was used to test differences between controls, PCS– and PCS+ patients at *p<0.05* significance, with Student’s *t* test for continuous variables, Fisher’s exact or χ^2^ test for categorical variables and Mann-Whitney *U* test for ordinal variables. Permutation-based non-parametric two-sample testing was used to access significant group differences in graph metrics (10,000 permutations), controlled for motion (adding age, gender and SCL as confounds overall does not change the results) and corrected for multiple comparisons when required. Two kinds of multiple comparisons correction were used, the conventional FDR threshold (*q <0.05*) and a less conservative false-positive correction, *p<1/N* (where here *N* means the number of comparisons, equivalent to expect less than one false positive per variable tested) [43]. To test group effects on mass pairwise measures, i.e. functional connectivity (*w_ij_*) or edge betweenness centrality (see supporting information File S1), we used network-based statistic [53]. The strength of the group difference (i.e. effect size) was assessed using Cohen’s *d* score. Additionally, we explored associations between symptom severity and graph theory properties using Pearson’s correlation coefficient over mTBI patients (at the subacute and late phases after the injury and independently of the presence of PCS) and controls.

Because of the large number of measures computed per subject and region, we chose to use a representation introduced recently, namely a connectogram, to display regional statistics [54]. The connectogram is represented in a circular fashion allowing to project complex graph architectures (or connectomes) in a 2-dimensional plane (circos.ca/). The BrainNet Viewer software was used to map the anatomical distribution of graph theory measures within the MNI standard space (www.nitrc.org/projects/bnv) [55].

## Results

### Clinical Evaluation

Seventeen PCS+ and 38 PCS– were identified on the basis of their symptoms at the late phase. The GCS did not differ between PCS+ and PCS– patients: all patients had a GCS of 15, except for one PCS+ patient ad one PCS– patient who both presented a GCS of 14. The main origins of TBI were aggressions, followed by falls, and work and sports accidents. Compared with healthy volunteers, significant higher values in symptom severity were observed in both PCS+ and PCS– patients at the subacute phase. PCS+ patients had significantly greater symptom severity than PCS– patients. At the late phase, PCS+ patients had more severe symptoms than both controls and PCS– patients. No significant between-group difference was found for age, gender, SCL and origins of injury (Table 1).

### Overall Graph Characteristics

**Figure 1:**
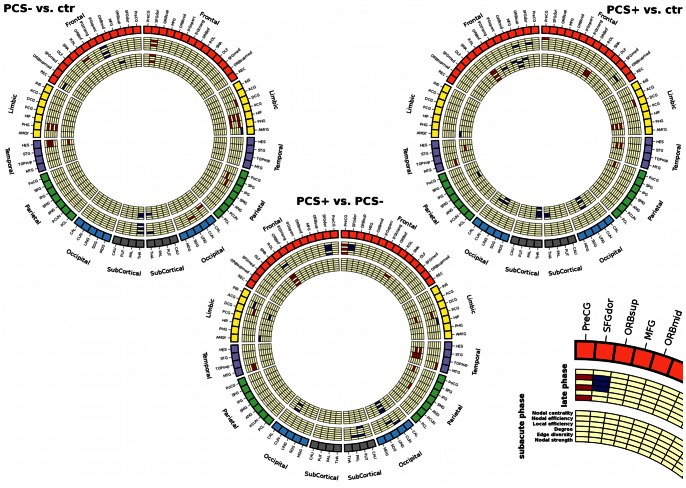
Association of modularity (left) and local efficiency of superior frontal regions (right) with symptom severity in mTBI patients at the late phase.

All networks studied, across individuals, presented a small world behavior characterized by a modular, hierarchical, assortative and non-random topology. Significant differences were found between groups for modularity, which was significantly lower in PCS+ patients than in PCS– patients at the late phase (*p* = 0.03, Table 1). This suggested that networks were less partitioned into communities in PCS+ patients. Moreover, we observed a small, but significant, negative relation between modularity and symptom severity in mTBI patients at 6 months after injury only (r = −0.33, p = 0.013; Figure 1). There were no other significant differences in overall properties.

### Regional Analysis

**Figure 2:**
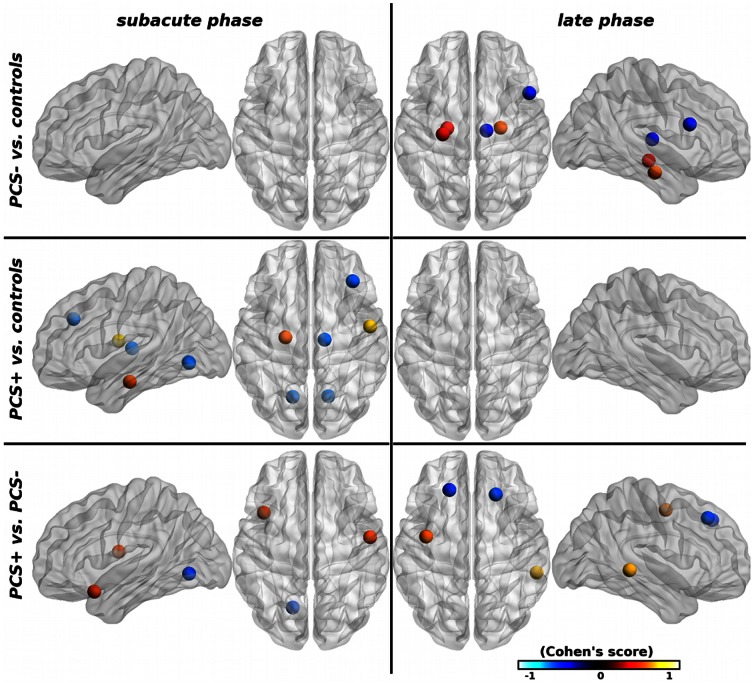
Statistical connectograms of differences between mTBI patients and controls, showing significant effect in regional characteristics at the subacute and late phases after the injury at *p<0.05* (uncorrected). The outermost ring shows brain regions arranged by lobe and subcortical structures and further ordered anterior-to-posterior (color map is lobe-specific). Bottom right: legend of the representation of regional metrics in the connectogram. Within the circular structure representing the brain parcellation, two sets of seven circular heat maps (or rings) are shown. Each set encodes for the subacute and late phases after the injury respectively, while each heat map encodes for regional graph theory properties (basic and topological) associated with the corresponding parcellation. Proceeding inward towards the center of the circle, these measures are: nodal centrality and efficiency, local efficiency, degree, edge diversity and strength. Red (resp. blue) color represents positive (resp. negative) effect.

Statistical connectograms revealed complex patterns of differences in regional characteristics within mTBI patients compared with controls (Figure 2). The list of regions where graph theory properties differed significantly between groups is presented in Table S2. Of note, regional effects did not survive FDR correction. PCS+ patients showed greater differences when compared with controls than when compared with PCS– patients. Predominant differences were found within limbic structures and in the occipital, temporal and frontal lobes.

#### Basic measures

**Figure 3:**
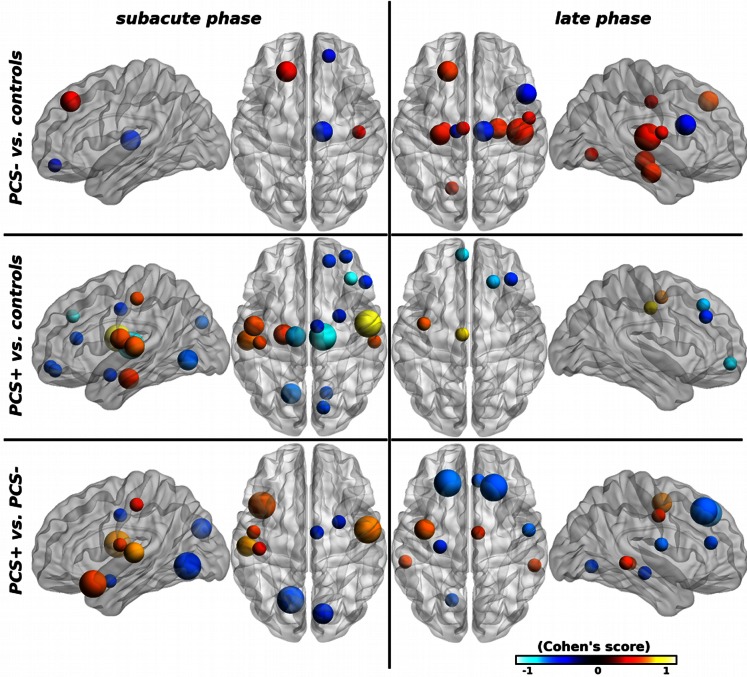
Brain regions with significant group differences in basic graph properties (*s* and *d_e_*) between mTBI patients and controls at p<0.05 (uncorrected). The node size indicates the number of basic properties with significant group differences. The nodal regions are located according to their centroid stereotaxic coordinates. Nodal color code for the average size effect, hot (resp. cold) colors represent increased (resp. decreased) properties.

Compared with healthy volunteers, mTBI patients presented differences in nodal strength and edge diversity, i.e. sum and variance of node connections, respectively. Significant differences were found in PCS– patients compared with controls only at the late phase after the injury, with an increase in the limbic system (bilateral parahippocampal gyrus and left hippocampus), a decrease in the right inferior frontal operculum and right thalamus (Figure 3). Compared with controls, PCS+ patients presented significant differences only at the subacute phase, characterized by increased connectivity in the left parahippocampal gyrus and the right rolandic operculum, and decreased connectivity in bilateral lingual gyrus, middle frontal gyrus, and the right thalamus. At the subacute phase, the basic measures were statistically higher in PCS+ than in PCS– patients in the right rolandic operculum and left temporal pole, and lower in the left lingual gyrus (Figure 3). At the late phase, the basic properties were higher in the left precentral gyrus and right middle temporal gyrus, and lower in bilateral superior frontal gyri. No significant between-group difference was found for functional connectivity.

#### Topological properties

**Figure 4:**
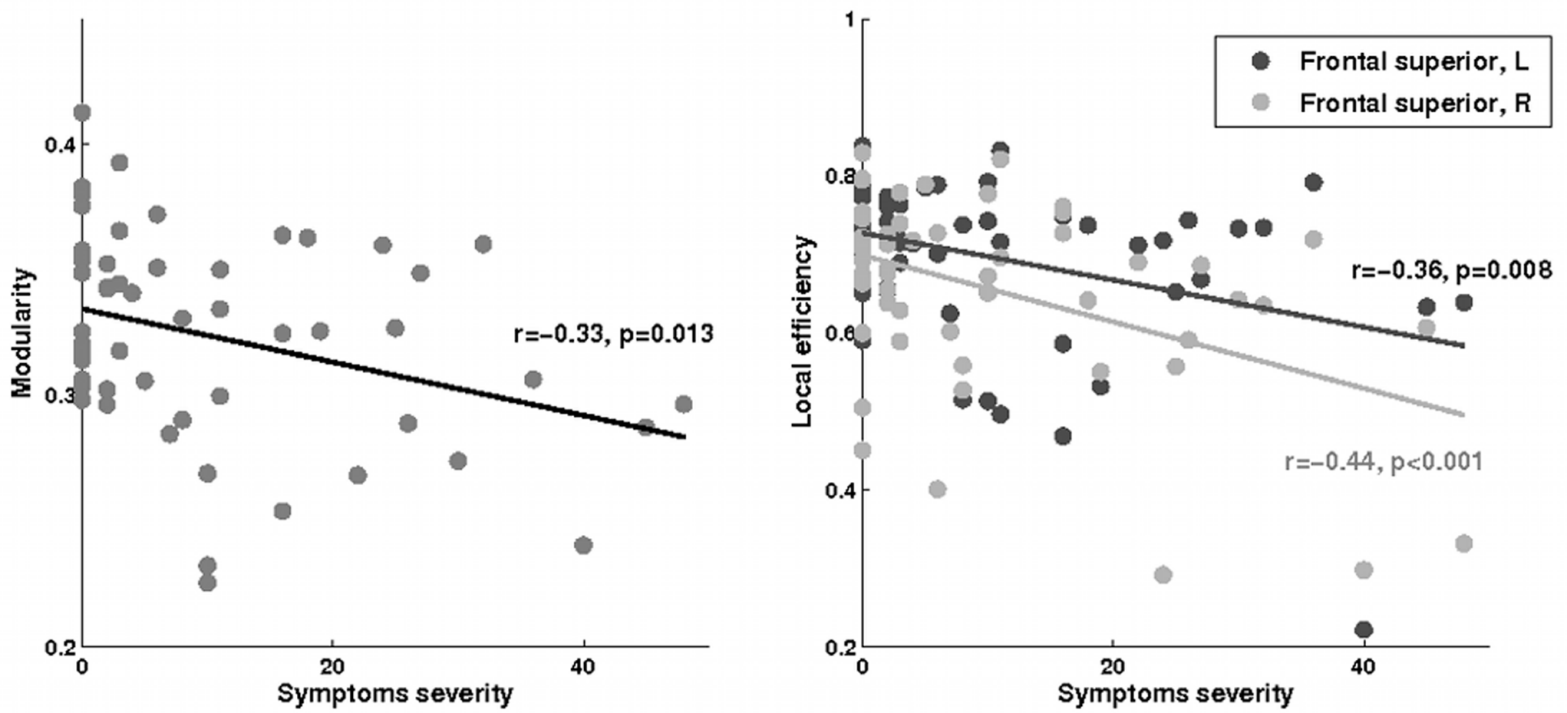
Brain regions with significant group differences in topological graph properties (*k*, *E_l_*, *E_n_* and *bc*) between mTBI patients and controls at p<0.05 (uncorrected). The node size indicates the number of topological properties with significant group differences. The nodal regions are located according to their centroid stereotaxic coordinates. Nodal color code for the average size effect, hot (resp. cold) colors represent increased (resp. decreased) properties.

Patients presented more differences in topological properties than in connectivity measures. In PCS– patients compared with controls differences were mostly found at the late phase in the limbic system with an increase in properties (Figure 4). In PCS+ patients at the subacute phase compared with controls, significant increase was observed in temporal regions and decrease in occipital and frontal lobes. Differences tended to normalize at the late phase with only few of them remaining significant (predominately the frontal lobe, including the right middle and superior frontal gyri and left middle fronto-orbital gyrus). The direct comparison between PCS+ and PCS– patients showed that temporal regions presented increased values in PCS+ patients (left superior temporal gyrus and left temporal pole) at the subacute phase, whereas no differences were observed at late phase. In contrast the frontal regions showed the opposite pattern with no difference at the early phase and significant differences at the late phase. When comparing with control subjects, the thalamus had lower topological properties in both PCS+ and PCS– patients at the subacute phase that remained slightly different at the late phase in PCS– patients only (Figure 4). No significant difference was observed in edge centrality.

#### Associations with symptom severity

In mTBI patients at the late phase, symptom severity correlated positively and significantly (even if the effect was small) with local properties, especially the local efficiency, in bilateral superior frontal gyrus (Figure 1). In healthy volunteers, symptom severity correlated negatively with regional properties in limbic regions including the amygdala, hippocampus and parahippocampal gyrus (Table 2).

**Table 2:** Regions with significant graph properties associations with symptom severity (corrected, p<1/N).

*Brain regions*		*Healthy volunteers*			*mTBI at late phase*		
			*r*	*p*		*r*	*p*
**Frontal**	Superior frontal, L				*E_l_*	−0.36	0.008
	Superior frontal, R				*s*	−0.38	0.005
					*k*	−0.37	0.006
					*E_l_*	−0.44	<0.001
					*E_n_*	−0.43	0.001
**Limbic**	Amygdala, R	*k*	−0.48	0.003			
		*E_n_*	−0.51	0.002			
	Hippocampus, R	*s*	−0.45	0.007			
		*d_e_*	−0.47	0.006			
		*k*	−0.49	0.004			
		*E_l_*	−0.53	0.001			
		*E_n_*	−0.51	0.002			
	Parahippocampus, L	*k*	−0.47	0.005			
	Parahippocampus, R	*s*	−0.45	0.007			
		*d_e_*	−0.46	0.006			
		*k*	−0.53	0.001			
		*E_n_*	−0.56	<0.001			
**Subcortical**	Putamen, L				*s*	−0.34	0.011
					*E_n_*	−0.36	0.007

Abbreviations: *d_e_*, edge diversity; *E_l_*, local efficiency; *E_n_*, nodal efficiency; *k*, degree; L, left; R, right; *s*, strength. Definition of basic (*s* and *d_e_*) and topologic (*k*, *E_l_* and *E_n_*) measures can be found in the subsection “*Characteristics of brain network organization using graph theory*” of the Materials and Methods section and in the supporting information File S1, respectively.

## Discussion

We examined longitudinal mTBI-induced functional changes in a global brain network using resting-state fMRI and graph theory in mTBI patients developing or not persistent PCS. Compared with both controls and patients who did not develop PCS, PCS+ patients presented strengthened and wide organizational characteristic changes. Graph properties were increased in temporal regions, predominantly at the subacute phase after the injury, and decreased in frontal regions, mainly at the late phase. These changes were functionally significant in PCS+ patient as they correlated with symptom severity in the frontal lobes.

In the present study, about a third of mTBI patients developed a PCS, a number which is in the upper limit of the reported prevalence of PCS after brain trauma, which is estimated at 15 to 30% [56]. Previous studies investigating brain networks following mild TBI have revealed alterations of network properties including reduced number and strength of connections in the default mode network and increased connections between the default mode network and the prefrontal cortex [25], [27], [28], [31], reduced interhemispheric connectivity in the primary visual cortex, hippocampus and dorsolateral prefrontal cortex networks [26], as well as increased overall strength [30]. Over the course of recovery, return to baseline of functional changes were observed in moderate to severe TBI patients [30], while persistent alterations were observed in symptomatic mTBI patients [31]. Overall, our observations on basic measures (connectivity strength and diversity) are in accordance with the literature. The novelty of this set of data is mostly related to the demonstration of increased connectivity in temporal regions and decreased connectivity in frontal regions in mTBI patients with PCS.

Basic connectivity alterations were accompanied by changes in the topological pattern including degree and nodal/local efficiency, and centrality. Pronounced loss of topological properties was found in frontal regions predominant at the late phase after the injury in PCS+ patients, while an increase was observed in temporal regions mainly at the subacute phase, compared with both PCS– patients and controls. The thalamus had lower topological efficiency in mTBI patients with or without PCS at the subacute phase. The efficiency of superior frontal regions was associated with symptom severity. Taken together, our results demonstrate PCS–specific alterations in the topological connectivity pattern following mild TBI and their evolution over time. The loss of connectivity in frontal and thalamic regions was concordant with recent reports [57], [58]. Only one study has investigated alterations of network topology longitudinally from 3 to 6 months post injury showing changes in degree, i.e. the number of connections between nodes, and increased smallworldness, with the strength but not the number of network connections returning to controls values [30]. The fact that almost no differences were found in PCS+ patients at the late phase compared with control subjects was explained by an increase in graph properties in the limbic system in PCS+ patients and in frontal regions in PCS– patients that reduced the effect size (see Figure S1). The localization of functional connectivity changes was clinically relevant, since these brain structures play an essential role in executive functions, cognitive control, working memory, attention and concentration that are typically affected in PCS patients [59], [60].

Functional changes may result from damage to white matter fiber tracts. Indeed, numerous works have shown the presence of axonal injury in patients with mTBI [61], [62]. More recently, it has been shown that structural changes in these patients correlated with neuropsychological impairments [63] and subjective complaints [7]–[9]. In addition when performing a choice-reaction task, mTBI patients showed greater deactivation within the default mode network and the magnitude of deactivation correlated with the amount of axonal injury in the adjacent corpus callosum further pointing to a link between anatomical damages and brain dysfunction [28]. Interestingly, in our previous studies [9], [10], mTBI patients (although slightly more affected) with PCS had greater and wider structural damage in fiber tracts containing thalamo-cortical as well as frontal connections, which coincides with the functional alterations shown in this work. Increased functional connectivity in the temporal lobe may be a compensatory process to the frontal damages, but further investigations are needed to verify such hypothesis.

Our analysis has some limitations. Here, we used the classical cumulative thresholding procedure to explore topological patterns, however such an approach provided few information regarding weak connections (cumulative thresholding have natural tendency to emphasize strong connections by construction). An alternative approach, the windowed thresholding, was recently proposed, which builds graphs by retaining connections within a range of thresholds [64], [65]. Such process provides insights into the examination of independent sets of connections (e.g. exploring the topology of the weakest connections). Further investigation is needed to assess whether a specific connection range is affected in mTBI population and related PCS. Another limitation came from the criteria defining PCS in mTBI patients. Indeed no consensus exists in the definition of PCS [66]. Unlike in our previous studies [9], [10], we used the DSM-IV classification [34], which is more restrictive than the ICD-10 criteria, including objective cognitive impairment [67]. It would be of great interest to examine to which extent PCS definitions differ and how this affects structural as well as functional integrity.

The detection of changes in functional connectivity in PCS+ patients may help improving diagnosis classifications by providing potential predictive biomarkers of neurobehavioral disorders consecutive to mild TBI that may contribute to reduce the heterogeneity of classifications [68], [69].

### Conclusions

Alterations of functional connectivity patterns were found in mTBI patients that evolved over time. Overall, mTBI patients showed increased connectivity in the limbic system after the trauma, whereas mTBI patients with PCS showed specific early thalamic and temporal and late frontal changes after the injury. The results highlight the relationship between functional brain network integrity and PCS. Further investigation remains to be conducted to better understand the relationship between the structural and functional alterations.

## Supporting Information

Figure S1 **Regional group differences in topological properties between mTBI patients and controls (unthresholded).** The nodal regions are located according to their centroid stereotaxic coordinates. Nodal color and size code for the average size effect over properties, hot (resp. cold) colors represent increased (resp. decreased) properties.
(TIFF)

Table S1 **The Rivermead Postconcussion Symptoms Questionnaire.**
(PDF)

Table S2 **Regions with significant group effects on basic and topological graph properties (uncorrected, p<0.05).**
(PDF)

Text S1 **Graph theory measures.**
(DOC)
