# Correction: Specific and Evolving Resting-State Network Alterations in Post-Concussion Syndrome Following Mild Traumatic Brain Injury

**DOI:** 10.1371/annotation/fd9f9796-b42d-480d-b9f4-0adfbb919148

**Published:** 2013-10-25

**Authors:** Arnaud Messé, Sophie Caplain, Mélanie Pélégrini-Issac, Sophie Blancho, Richard Lévy, Nozar Aghakhani, Michèle Montreuil, Habib Benali, Stéphane Lehéricy

The titles and legends of multiple figures were incorrectly switched. The title and legend currently appearing with Figure 1 belong with Figure 4, the title and legend currently appearing with Figure 2 belong with Figure 1, the title and legend currently appearing with Figure 3 belong with Figure 2, and the title and legend currently appearing with Figure 4 belong with Figure 3. The images themselves are in correct order.

In addition, there was an error in the first sentence of the paragraph titled "Associations with symptom severity" in the "Regional Analysis" section of the Results. The correct sentence is: "In mTBI patients at the late phase, symptom severity correlated negatively and significantly (even if the effect was small) with local properties, especially the local efficiency, in bilateral superior frontal gyrus (Figure 1)." 

