# Supplementary figures and images for: Specific and Evolving Resting-State Network Alterations in Post-Concussion Syndrome Following Mild Traumatic Brain Injury
Source: PLoS One. 2013 Jun 6;8(6):e65470. doi: 10.1371/journal.pone.0065470 (PMC3675039; doi:10.1371/journal.pone.0065470)

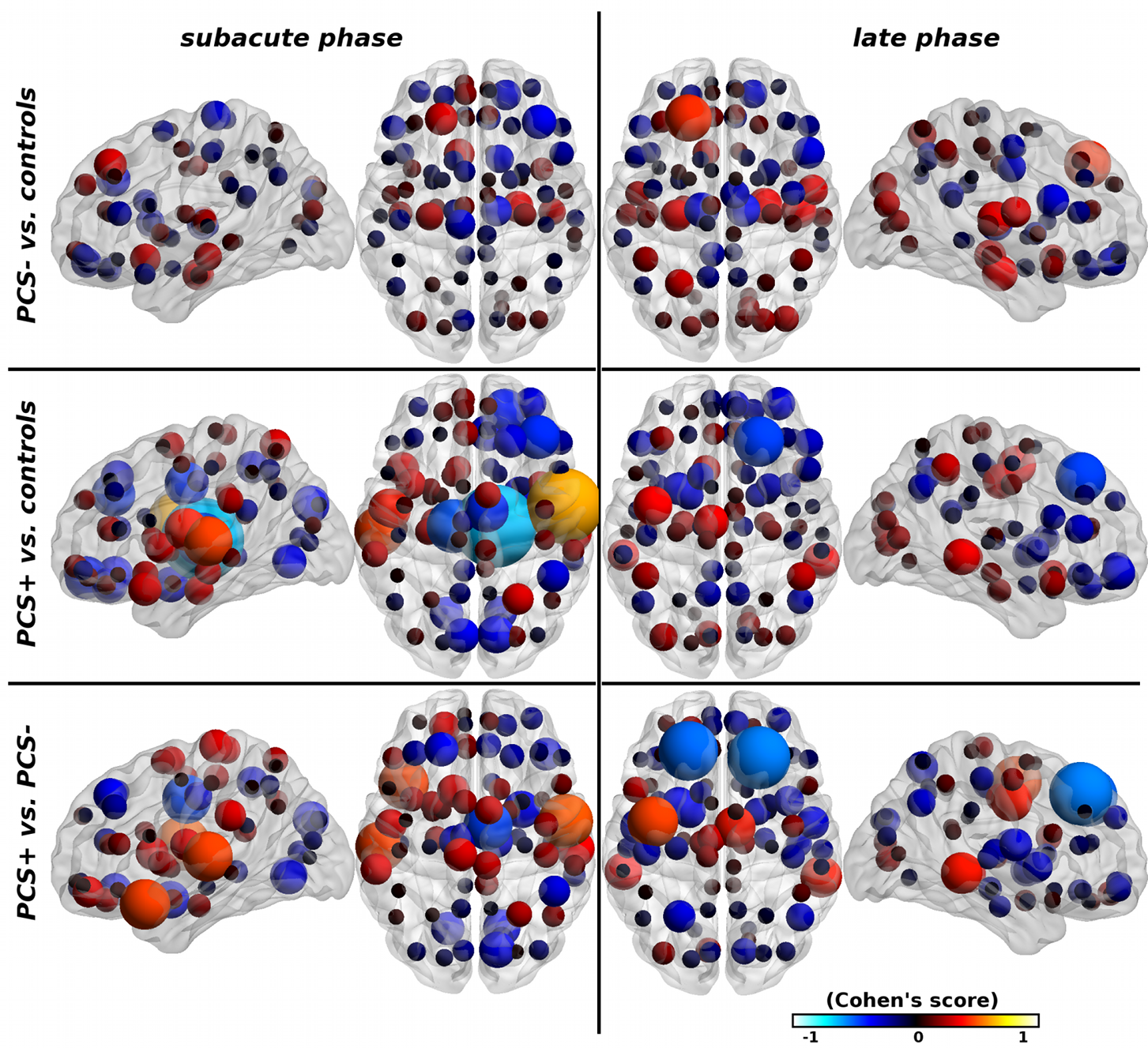

Supplement: Figure S1 — Regional group differences in topological properties between mTBI patients and controls (unthresholded). The nodal regions are located according to their centroid stereotaxic coordinates. Nodal color and size code for the average size effect over properties, hot (resp. cold) colors represent increased (resp. decreased) properties. (TIFF) [file pone.0065470.s001.tiff]
