# Supplementary material for: Specific and Evolving Resting-State Network Alterations in Post-Concussion Syndrome Following Mild Traumatic Brain Injury
Source: PLoS One. 2013 Jun 6;8(6):e65470. doi: 10.1371/journal.pone.0065470 (PMC3675039; doi:10.1371/journal.pone.0065470)
Supplement: Table S1 — The Rivermead Postconcussion Symptoms Questionnaire. (PDF) [file pone.0065470.s002.pdf]

Table S1: The Rivermead Postconcussion Symptoms Questionnaire.

Symptoms	***Controls***	***Subacute phase***		***Late phase***	
		PCS-	***PCS+***	***PCS-***	***PCS+***
Headaches	0.18 (0.46)	1.40 (1.50)	2.20 (1.3)0	0.68 (1.20)	1.80 (1.40)
Feelings of dizziness	0 (0)	0.39 (0.86)	1.80 (1.40)	0.05 (0.23)	0.88 1.40)
Nausea and/or vomiting	0.03 (0.17)	0.21 (0.81)	1.10 (1.20)	0.13 (0.66)	0.59 (1.00)
Noise sensitivity	0 (0)	0.61 (1.10)	2.10 (1.60)	0.34 (0.85)	1.90 (1.60)
Sleep disturbance	0.26 (0.62)	1.40 (1.70)	2.70 (1.50)	0.47 (1.00)	3.10 (1.20)
Fatigue. tiring more easily	0.41 (0.74)	1.40 (1.60)	3.10 (1.10)	0.34 (0.85)	3.00 (1.10)
Being irritable. easily angered	0.21 (0.73)	0.71 (1.20)	2.40 (1.30)	0.24 (0.79)	1.90 (1.40)
Feeling depressed or tearful	0.06 (0.34)	0.61 (1.20)	1.90 (1.50)	0.05 (0.23)	2.00 (1.40)
Feeling frustated or impatient	0.21 (0.54)	0.68 (1.20)	2.40 (1.50)	0.11 (0.39)	2.10 (1.40)
Forgetfulness. poor memory	0.44 (0.86)	0.53 (1.00)	2.00 (1.30)	0.32 (0.84)	1.80 (1.40)
Poor concentration	0.56 (0.89)	0.84 (1.20)	2.50 (1.20)	0.53 (1.00)	1.90 (1.30)
Slowness	0.12 (0.41)	0.39 (0.97)	1.80 (1.30)	0.21 (0.62)	1.40 (1.30)
Blurred vision	0.03 (0.17)	0.34 (0.97)	1.50 (1.60)	0.13 (0.58)	1.20 (1.70)
Light sensitivity	0 (0)	0.26 (0.86)	1.60 (1.70)	0.03 (0.16)	1.10 (1.50)
Double vision	0 (0)	0 (0)	0.76 (1.40)	0 (0)	0.18 (0.73)
Restlessness	0.03 (0.17)	0.05 (0.32)	1.20 (1.60)	0 (0)	0.59 (1.10)
