# Supplementary material for: Specific and Evolving Resting-State Network Alterations in Post-Concussion Syndrome Following Mild Traumatic Brain Injury
Source: PLoS One. 2013 Jun 6;8(6):e65470. doi: 10.1371/journal.pone.0065470 (PMC3675039; doi:10.1371/journal.pone.0065470)
Supplement: Table S2 — Regions with significant group effects on basic and topological graph properties (uncorrected, p<0.05). (PDF) [file pone.0065470.s003.pdf]

Table S2: Regions with significant group effects on basic and topological graph properties (uncorrected, $p<0.05$). Bold indicates significance at $p<1/N$.

Brain regions		***Subacute phase***			***Late phase***		
		**PCS-/ctr**	**PCS+/ctr**	**PCS+/PCS-**	**PCS-/ctr**	**PCS+/ctr**	**PCS+/PCS-**
Frontal	Inferior frontal triangular, R		E_l 0.624				
	Inferior frontal opercular, R				s 0.525<br>k 0.508<br>bc 0.492		
	Middle frontal, R		d_e 0.652<br>**E_l 0.999**			E_l 0.647	
	Middle frontal orbital, L					**E_l 0.871**	
	Middle frontal orbital, R	k 0.460	E_n 0.639				
	Superior frontal, L	k -0.473<br>bc -0.491			**k -0.597**<br>**E_n -0.697**		k 0.659<br>**E_l 0.842**<br>**E_n 0.736**
	Superior frontal, R					**E_l 0.836**	s 0.638<br>k 0.680<br>E_l 0.731<br>**E_n 0.735**
	Superior frontal orbital, R		E_n 0.639				
	Precentral, L					bc -0.699	s -0.664<br>**k -0.762**<br>E_n -0.634
	Rolandic operculum, L		k -0.708<br>E_n -0.619	E_n -0.619			
	Rolandic operculum, R		**s -0.804**<br>**k -1.041**<br>**E_n -1.013**<br>**bc -0.857**	s -0.592<br>**k -0.775**<br>**E_n -0.771**<br>bc -0.637	E_n -0.493		E_l 0.698
Limbic	Amygdala, R		bc 0.603	bc 0.607			
	Anterior cingulum, R						E_l 0.673
	Middle cingulum, L				bc -0.474	**bc -0.873**	
	Middle cingulum, R		E_l 0.582	k 0.586			k -0.606
	Hippocampus, L				s -0.492<br>k -0.540<br>**E_n -0.580**		bc 0.586
	Parahippocampus, L		s -0.618<br>k -0.593<br>E_n -0.561		s -0.476<br>E_n -0.509		
	Parahippocampus, R				s -0.634<br>k -0.567<br>**E_n -0.596**		
Temporal	Heschl, R	k -0.476			k -0.556<br>**E_l -0.606**<br>E_n -0.528		
	Superior temporal, L		k -0.686<br>**E_n -0.734**	**k -0.724**<br>**E_n -0.814**			
	Superior temporal, R		E_l -0.663				
	Superior temporal pole, L			d_e -0.597<br>k -0.696<br>E_l -0.679<br>E_n -0.574			
	Middle temporal, L						bc 0.604
	Middle temporal, R						**ed -0.765**<br>k -0.600
Parietal	Postcentral, L		E_l -0.652	E_l -0.512			
Occipital	Cuneus, R		k 0.670	k 0.605<br>bc 0.576			
	Lingual, L		s 0.654<br>k 0.664<br>**bc 0.777**	s 0.581<br>k 0.619<br>E 0.713<br>bc 0.570	E_l -0.506		E_l 0.628
	Lingual, R		s 0.649<br>k 0.642				
Subcortical	Thalamus, L		**E_l 0.823**<br>**E_n 0.795**		E_n 0.466		
	Thalamus,R	E_l 0.612<br>E_n 0.509	s 0.666<br>**k 0.774**<br>**E_l 0.927**<br>**E_n 0.939**		s 0.473<br>**k 0.560**<br>E_n 0.519		

Abbreviations: ctr, controls; d_e, edge diversity; E_l, local efficiency; E_n, nodal efficiency; k, degree; L, left; PCS, post-concussion syndrome; R, right; s, strength. Definition of basic (s and d_e) and topographic (k, E_l, E_n and bc) measures can be found in the subsection "*Characteristics of brain network organization using graph theory*" of the Materials and Methods section and in the supplementary information section, respectively.
