# Supplementary material for: Specific and Evolving Resting-State Network Alterations in Post-Concussion Syndrome Following Mild Traumatic Brain Injury
Source: PLoS One. 2013 Jun 6;8(6):e65470. doi: 10.1371/journal.pone.0065470 (PMC3675039; doi:10.1371/journal.pone.0065470)
Supplement: Text S1 — Graph theory measures. (DOC) [file pone.0065470.s004.doc]

**Supporting Information: Graph theory measures**

Topological properties detect several aspects of brain networks such as its smallworldness [1], its functional integration and segregation using the efficiency measures [2-4], centrality [5], modularity [6], hierarchy [4,7], assortativity [6], and resilience to insult [3,8]. Here we introduce the graph theory measures investigated in the study. We consider a network *G* composed of a set of *N* nodes and a set of *M* edges.

*Smallworld properties*

We used a smallworldness measure derived from the characteristic path length of a graph and its clustering coefficient [1,9]. The characteristic path length *L* of a graph is the average of the shortest path lengths between all pairs of nodes, where the shortest path length between two nodes corresponds to the minimum number of distinct edges required to link these nodes. The clustering coefficient *C* is the average of the clustering coefficients of all nodes, where the clustering coefficient of a node is the ratio between the actual number of edges among the node’s neighbours and the largest number of possible connections within this neighbourhood.

Random and lattice networks represent extremes of network topology from a totally disordered network to a totally regular network. Typically, a random network is characterized by small *L* and *C* values compared with a regular network. Smallworld networks are located between the two extreme configurations (random and lattice) and are characterized by a small *L* value (comparable to random networks) and a high *C* value (comparable to lattice networks). Smallworldness, *σ*, is formulated as follows:

$$\sigma=\frac{C/C_{r}}{L/L_{r}},$$

where *C_r_* (respectively, *L_r_*) is the average clustering (respectively, path length) value obtained for matched random networks (i.e. networks with the same size and density as the analyzed network) [10]. A network will therefore be considered as having the smallworld behavior if *σ* > 1 [11].

*Efficiency and centrality*

The global efficiency, *E_g_*, is defined as the average nodal efficiency (*E_n_*) [2]:

$$E_{g}=\frac{1}{N}\sum E_{n}=\frac{1}{N\left(N-1\right)}\sum\frac{1}{L_{ij}},$$

where *L_ij_* corresponds to the path length between nodes *i* and *j*. The local efficiency, *E_l_*, is defined as the average global efficiency of subnetworks:

$$E_{l}=\frac{1}{N}\sum E_{g}\left(G_{i}\right),$$

where *G_i_* is the subnetwork composed of the nearest neighbours of *i* (i.e. the set of nodes directly linked to *i*).

Measure of centrality is related to the idea that some nodes are committed in many short paths and play an central role of information flow within a network [5]. Here we used betweenness centrality, which is defined as the fraction of all shortest paths in the network that pass through a given node.

$$bc_{i}=\frac{1}{\left(N-1\right)\left(N-2\right)}\sum\frac{\rho_{jk|i}}{\rho_{jk}},$$

where *ρ_jk_* is the number of shortest paths between *j* and *k*, and *ρ_jk|_*_i_ is the number of shortest paths between *j* and *k* that pass through *i*. The notion of betweenness centrality can be naturally extended to edges.

*Modularity and hierarchy*

Networks are often partitioned into a set of modules or communities having dense interconnected nodes and less communication with nodes of the others modules. The measure of modularity [6] is defined as

$$Q=\sum\left[e_{uu}-\left(\sum e_{uv}\right)^{2}\right],$$

where the network is subdivided into a set of *M* modules, and *e_uv_* is the proportion of links that connect nodes in module *u* with nodes in module *v*. Typically, a network with $Q$ value greater than 0.2 is considered as having a modular structure.

Networks may also display a hierarchical architecture, which can be characterized by the parameter *β* quantifying the power-law relationship between the clustering coefficient and the degree [7]: *C* ∼ *k*^−^*^β^*. Hierarchical networks are characterized by a modular architecture at multiple scales, all encapsulated within one another.

*Assortativity and robustness*

Assortativity measures the preference of nodes to connect to other nodes of similar degree (assortative, *r* > 0, or dissortative, *r* < 0), and is defined as [6]:

$$r=\frac{\frac{1}{M}\sum k_{i}k_{j}-\left[\frac{1}{\text{2M}}\sum\left(k_{i}+k_{j}\right)\right]^{2}}{\frac{1}{M}\sum\left(k_{i}^{2}+k_{j}^{2}\right)-\left[\frac{1}{\text{2M}}\sum\left(k_{i}+k_{j}\right)\right]^{2}}.$$

Robustness quantifies the network resilience to insult either targeted or random. Targeted attacks remove nodes in the descending order of degree, while random attacks remove nodes at random. At each attack, the size of the largest connected component is calculated. Robustness is defined as the integral of the size of the largest connected component (group of nodes in the network that are all reachable from each other), function of the number of nodes removed [12].

**References**

1. Humphries M, Gurney K (2008) Network ’small-world-ness’: A quantitative method for determining canonical network equivalence. PLoS ONE 3: e0002051.
2. Latora V, Marchiori M (2001) Efficient behavior of small-world networks. Physical Review Letters 87: 198701-4.
3. Achard S, Bullmore E (2007) Efficiency and cost of economical brain functional networks. PLoS Computational Biology 3: e17.
4. Bassett DS, Brown JA, Deshpande V, Carlson JM, Grafton ST (2011) Conserved and variable architecture of human white matter connectivity. Neuroimage 54: 1262-79.
5. Freeman L (1977) A set of measures of centrality based on betweenness. Sociometry 40: 35-41.
6. Newman MEJ (2006) Modularity and community structure in networks. Proceedings of the National Academy of Sciences of the United States of America 103: 8577-82.
7. Ravasz E, Barabási AL (2003) Hierarchical organization in complex networks. Physical Review E 67: 026112.
8. Lynall ME, Bassett D, Kerwin R, McKenna P, Kitzbichler M, Muller U, et al (2010) Functional connectivity and brain networks in schizophrenia. The Journal of Neuroscience 30: 9477-87.
9. Watts DJ, Strogatz SH (1998) Collective dynamics of ’small-world’ networks. Nature 393: 440-2.
10. Maslov S, Sneppen K (2002) Specificity and stability in topology of protein networks. Science 296: 910-13.
11. Strogatz SH (2001) Exploring complex networks. Nature 410: 268-76.
12. Achard S, Salvador R, Whitcher B, Suckling J, Bullmore E (2006) A resilient, low-frequency, small-world human brain functional network with highly connected association cortical hubs. The Journal of Neuroscience 26: 63-72.
